# Evaluating Immune Activation Feasibility in Pancreatic Ductal Adenocarcinoma via Oxygen Bubble-Induced Anti-Vascular Therapy

**DOI:** 10.3390/pharmaceutics17050645

**Published:** 2025-05-13

**Authors:** Tzu-Yun Chiu, Yi-Jia Zho, Yi-Ju Ho

**Affiliations:** 1 Department of Biological Science and Technology, College of Engineering Bioscience, National Yang Ming Chiao Tung University, Hsinchu 30010, Taiwan; 2 Institute of Molecular Medicine and Bioengineering, College of Engineering Bioscience, National Yang Ming Chiao Tung University, Hsinchu 30010, Taiwan; 3 Center for Intelligent Drug Systems and Smart Bio-Devices (IDS2B), National Yang Ming Chiao Tung University, Hsinchu 30010, Taiwan

**Keywords:** vascular disruption, antigen, ultrasound, bubble cavitation, hypoxia

## Abstract

**Background/Objectives:** Anti-vascular therapy presents a potential strategy for activating anti-tumor immunity. Disrupted vascular debris provides effective antigens that activate dendritic cells, leading to subsequent immune responses. However, the resulting tumor hypoxia following vascular disruption may contribute to immune suppression, thereby hindering effective immune activation. Ultrasound-stimulated microbubble cavitation can locally disrupt tumor vessels through mechanical effects to achieve physical anti-vascular therapy. Therefore, this study designed oxygen-loaded nanobubbles (ONBs) to combine anti-vascular effects with local oxygen release under ultrasound stimulation. The feasibility of enhancing anti-tumor immune activation by alleviating tumor hypoxia was evaluated. **Methods:** A murine pancreatic subcutaneous solid tumor model was used to evaluate the efficacy of anti-vascular therapy-associated immunotherapy. **Results:** After ONB treatment, tumor perfusion was reduced to 52 ± 5%, which resulted in a subsequent 57 ± 11% necrosis and a 29 ± 4% reduction in hypoxia, demonstrating the anti-vascular effect and reoxygenation, respectively. However, subsequent immune responses exhibited no significant activation in intratumoral cytokine expression or splenic immune cell composition. Primary tumors exhibited a 15.7 ± 5.0% increase in necrosis following ONB treatment, but distant tumor growth was not significantly inhibited. **Conclusions:** These results highlighted a crucial issue regarding the complex correlations between vessel disruption, antigen production, oxygen delivery, hypoxia, and immunity when combining anti-vascular therapy with immunotherapy.

## 1. Introduction

Immunotherapy is recognized as a powerful strategy for cancer therapy. Immune checkpoint inhibitors, immunostimulants, and chimeric antigen receptor T cells are used to promote the anti-tumor immune cells for killing tumors. However, its efficacy is limited by the heterogeneous and immunosuppressive tumor microenvironment [1]. The dysfunctional and abnormal tumor vessels cause tumor hypoxia to recruit tumor-associated macrophages and regulatory T cells, inhibiting anti-tumor immunity [2,3].

Anti-vascular therapy is a targeted approach designed to disrupt abnormal tumor vessels [4]. Vascular disrupting agents selectively damage the fragile tumor vasculature, leading to nutrient deprivation and subsequent tumor growth inhibition [5]. Since the structure of tumor vessels would be disrupted, the debris from tumor vessels or cells might serve as effective antigens for activating anti-tumor immunity. However, the uneven vascular disruption-induced rim effect and the resulting tumor hypoxia significantly hinder immune activation [6]. Shen et al. reported that using vascular-disrupting agent CA4 nanomedicine to treat murine 4T1 tumor model showed no obvious increase in the CD4+ helper T cells, CD8+ cytotoxicity T cells, natural killer cells, and mature dendritic cells due to the tumor hypoxia [7]. Seth et al. combined the vascular disrupting agent DMXAA and immune adjuvant toll-like receptors agonists to treat murine B16-F10 melanoma [8]. When DMXAA or an immune adjuvant was used alone, tumor treatment efficacy was inhibited due to uneven vascular disruption and insufficient antigen production, respectively. In addition, the systemic side effects of vascular disrupting agents, such as cardiotoxicity, bowel ischemia, and hypotension, also limit the treatment dose during the anti-vascular therapy-associated immunotherapy [9,10].

Ultrasound (US)-stimulated bubble cavitation has been widely applied in localized drug delivery, as well as in tumor, brain, and cardiovascular treatments [11,12]. Microbubbles are multifunctional US contrast agents that can carry drugs or gases for localized release under US stimulation, thereby combining diagnostic and therapeutic applications [13]. The mean diameter of microbubbles typically ranges from 1 to 8 µm, allowing safe injection into the bloodstream without causing gas embolism [14,15]. Due to the gas core within microbubbles, the reflection of ultrasonic waves is enhanced, improving the contrast between blood flow and surrounding tissues, and thus facilitating US contrast imaging for diagnostic purposes. When stimulated by low-intensity US, microbubbles undergo expansion and contraction, generating stable cavitation effects that enhance vascular permeability, promote blood perfusion, and open the blood–brain barrier [16]. In contrast, when stimulated by high-intensity US, microbubbles produce inertial cavitation effects that generate violent mechanical forces to disrupt cell membranes or vessel walls [17]. This anti-vascular effect induced by microbubble cavitation has been investigated over 15 years [17,18,19]. Compared to vascular disrupting agents, microbubble cavitation offers a localized and physical approach to therapy, minimizing systemic side effects. The intensity and uniformity of the anti-vascular effect can be modulated by optimizing US parameters and microbubble treatment dosage [19,20].

The investigation into combining US alone or with microbubble treatment to promote immunotherapy has gradually increased over the past decade [12,21,22]. Based on the thermal and mechanical effects of US alone or microbubble cavitation, the mechanisms behind activating immune responses can be explained by the following reasons [23]. First, high-intensity focused ultrasound (HIFU) ablation or microbubble cavitation increases the production of effective antigens from tumor cell or vessel debris, promoting the maturation of dendritic cells and subsequent anti-tumor immune responses [24,25]. Second, the increased permeability of tumor vessels due to HIFU hyperthermia or microbubble cavitation enhances immune cell infiltration into tumors [26,27]. Third, reoxygenation induced by enhanced tumor perfusion under HIFU hyperthermia, microbubble cavitation, or oxygen-loaded microbubble treatment inhibits hypoxia-induced immunosuppression [28,29]. Abe et al. compared the anti-tumor immune activation induced by the thermal and mechanical effects of HIFU without microbubbles in murine breast cancer models [30]. The thermal effect of HIFU (50% duty cycle), which caused coagulation necrosis through heating, resulted in insufficient systemic anti-tumor immunity due to the denaturation of tumor proteins. In contrast, the mechanical effect of HIFU (2% duty cycle) induced boiling histotripsy to disrupt tumor cells without increasing temperature. The effective antigens from tumor cell debris significantly enhanced the infiltration of helper T cells and cytotoxic T cells into tumors. However, significant inhibition of distant tumor growth through systemic anti-tumor immunity was observed only when mechanical HIFU treatment was combined with the immune checkpoint inhibitor anti-PD-L1 antibody. These results suggest that although the mechanical effects induced by HIFU or microbubble cavitation can generate effective antigens to activate local immune responses within tumors, the induction of a systemic anti-tumor immune response remains insufficient.

Based on these mechanisms, our study proposed that oxygen-loaded nanobubble (ONB) treatment could serve as both a multifunctional gas carrier and an immune activator to enhance immunotherapy. The anti-vascular effect induced by ONB cavitation may generate effective antigens to activate immune cells. The local oxygen release from ONBs could compensate for tumor hypoxia caused by vascular disruption. Moreover, the adequate oxygen supply also supports immune cell functions, contributing to the anti-tumor effect. Compared to microbubbles, ONBs provide a longer in vivo lifetime and more easily penetrate into tissues through disrupted vessels to overcome tumor hypoxia [31,32,33]. The optimal fabrication of ONBs and HIFU parameters were investigated in a murine pancreatic ductal adenocarcinoma model. Intravital vascular disruption and hemorrhage were observed using the murine dorsal skinfold window chamber tumor model. The changes in tumor perfusion, necrosis, and vessel density were analyzed to determine the degree of the anti-vascular effect. Finally, intratumoral cytokine expression, splenic immune cell composition, and tumor growth were quantified to evaluate the feasibility of anti-vascular therapy-associated immunotherapy induced by ONB treatment. Since pancreatic ductal adenocarcinoma is classified as an immunologically “cold” phenotype, this study further focused on investigating whether the combination of anti-vascular effects and localized oxygen release could reprogram the immunosuppressive tumor microenvironment into an immunoactive state.

## 2. Materials and Methods

### 2.1. Fabrication of ONBs

The materials used for constructing ONBs included 1,2-distearoyl-sn-glycero-3-phosphocholine (DSPC, Avanti Polar Lipids, Alabaster, AL, USA), 1,2-distearoyl-sn-glycero-3-phosphoethanolamine-N-[methoxy(polyethyleneglycol)-2000] (DSPE-PEG2000, Avanti Polar Lipids), octafluoropropane (C_3_F_8_, Sindagases, Hsinchu, Taiwan), and oxygen (Sindagases). The detailed steps for fabricating ONBs followed our previous studies [34,35,36]. DSPC and DSPE-PEG2000 formed a lipid shell that encapsulated gas, subsequently forming bubbles. NBs encapsulated 100% octafluoropropane gas, whereas ONBs encapsulated a mixture of octafluoropropane and oxygen. The ratios of octafluoropropane to oxygen in the gas mixture were 50%, 60%, and 70% oxygen. The optimal oxygen ratios in the gas mixture for ONB fabrication were determined based on the size distribution, concentration, and dissolved oxygen (DO) levels of the ONBs.

The nano-sized distribution of ONBs was measured using dynamic light scattering (Nano-ZS90, Malvern Panalytical, Malvern, UK). To calculate the concentration of micro-sized ONBs for biosafety and dosage evaluation, a hemocytometer was used to quantify the number of diluted ONBs under an inverted microscope (Eclipse Ti2, Nikon, Tokyo, Japan). In this study, size distribution measured by dynamic light scattering and concentration quantified using a hemocytometer were analyzed.

The in vitro DO levels were evaluated before and after replacing the fresh ONB solution to determine whether the oxygen was contained within the ONBs or dissolved in the solution. After centrifugation at 2000 rcf for 1 min, ONBs floated to form a layer of bubble cake, and the lower supernatant was replaced with an equal volume of fresh phosphate-buffered saline (PBS). A DO meter (EZDO, Taichung, Taiwan) was used to measure 4 × 10^10^ NBs/mL for 5 min.

### 2.2. In Vitro Stability of ONBs

The contrast enhancement of ONBs under US imaging was monitored over time to evaluate their in vitro stability. PBS-diluted ONBs (1 × 10^7^ NBs/mL) were infused into a hollow cavity within a 2% agarose phantom maintained at 37 °C. A commercial US imaging system (CX50, Philips, Amsterdam, The Netherlands) connected to an 8 MHz imaging transducer (L12-3, Philips) was used to acquire ONB images. The contrast mode of US imaging was selected to clearly visualize ONB signals, and images were captured at different time points. The contrast images were analyzed to calculate the signal-to-noise ratio using MATLAB software (R2021, MathWorks, Natick, MA, USA). Signal-to-noise ratios at different time points were normalized to the 0 min value and expressed as stability%.

### 2.3. In Vitro Partial Pressure of Oxygen (pO_2_) Detection

The ability of oxygen release from ONBs triggered by sonication was demonstrated by detecting the partial pressure of oxygen (pO_2_). A gas-sealed vial containing 500 μL degassed PBS was injected with 50 μL of diluted ONBs (5 × 10^7^ NBs/mL). The vial was then sonicated for 3 s using an ultrasonic cleaner (D150H, YUANTUO, Taichung, Taiwan) to disrupt ONBs and release oxygen. An OxyLite™ system (Oxford Optronix, Abingdon, UK) linked to a pO_2_ probe (OxyLite NX pO_2_ Bare Fibre Sensor, Oxford Optronix) was inserted into the vial to measure pO_2_ levels in the ONB solution for 5 min. The detection of in vitro pO_2_ was used to verify the oxygen-carrying and -releasing capabilities of ONBs.

### 2.4. Cell Line and Animal Model

The murine pancreatic ductal adenocarcinoma (Panc02) cell line was used in this study. Panc02 cells were cultured in Dulbecco’s modified Eagle’s medium (CORNING, Corning, NY, USA) supplemented with 1% penicillin–streptomycin and 10% fetal bovine serum (CORNING). The animal experiments used C57BL/6 JNarl mice to establish the murine dorsal skinfold window chamber tumor model and subcutaneous solid tumor model. A total of seventy-five mice (male, 6–10 weeks old) were purchased from the National Laboratory Animal Center. All animal experiments were approved by the animal experiment Committee at National Yang-Ming Chiao Tung University (IACUC Consent No.: 111025A).

To establish the murine dorsal skinfold window chamber tumor model (n = 9), 1 × 10^6^ Panc02 cells in 50 μL were subcutaneously injected into the mice’s dorsal skinfold. After 6 days of growth, when the tumor diameter reached 3 mm, the window chamber kits were mounted on the murine dorsal skin. The window chamber was implanted according to the procedures described in [20,37]. To establish the subcutaneous solid tumor model (n = 66), 1 × 10^6^ Panc02 cells in 100 μL were subcutaneously injected into the mice’s leg. After 9 days of growth, when the tumor volume reached 40 mm^3^, the experiments were initiated. During the experiments, mice were anesthetized via inhalation with 2% isoflurane or via intraperitoneal injection with a 1:1 mixture of Rompun 2% (Rompun 20, Elanco, Taipei, Taiwan) and Zoletil 50 (Zoletil 50, Virbac, Taipei, Taiwan).

### 2.5. Observation of Anti-Vascular Effect Under Intravital Imaging System

**Figure 1:**
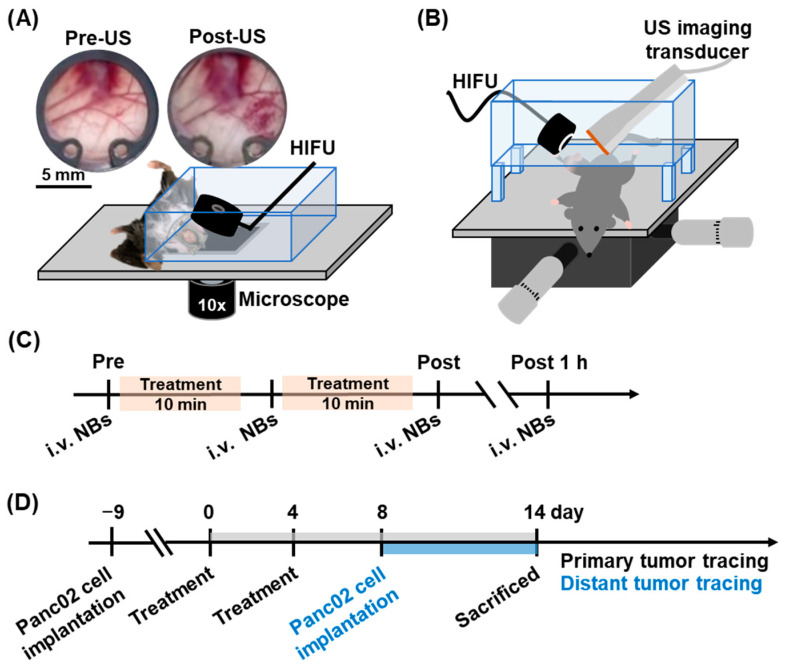
(**A**) Schematic illustration of an acousto-optical system with the murine window chamber tumor model. The post-US window chamber shows visualized hemorrhage. (**B**) Schematic illustration of US imaging-guided HIFU treatment system. (**C**) Flowchart of tumor perfusion tracing during the solid tumor treatment. (**D**) Flowchart of primary and distant tumor implantation and volume tracing.

To evaluate the vascular effects induced by NB treatment, an acousto-optical system integrating a 2 MHz HIFU transducer with an inverted microscope was used to record real-time intravital images (Figure 1A). The custom 2 MHz HIFU transducer (focal size: 1 mm in diameter × 3 mm in length; custom-made by National Cheng Kung University, Tainan, Taiwan) was calibrated the acoustic field and acoustic peak negative pressures by using a needle hydrophone (NH 1000, Precision Acoustics, Dorchester, UK) in an acrylic water tank filled with degassed and deionized water at 25 °C. A murine window chamber tumor model (n = 9) was positioned on the microscope stage of the acousto-optical system and i.v. injected with 5 × 10^7^ NBs. After 1 min circulation of NBs, a 2 MHz HIFU transducer was driven by a waveform generator (AFG1022, Tektronix, Beaverton, OR, USA) and a power amplifier (1020L, Electronics & Innovation, Rochester, NY, USA) to transmit a 1k cycle pulse with a peak negative pressure of 1.3, 2.0, and 2.6 MPa. Intravital images were acquired before and after 30 s of US stimulation to quantify intertissue hemorrhage using MATLAB software. To assess the degree of the anti-vascular effect, the hemorrhage percentage was calculated as the ratio of the hemorrhagic area to the total image area. Finally, the hemorrhage percentage under different acoustic pressures was analyzed to determine the optimal parameters for the 2 MHz HIFU.

### 2.6. Optimizing Treatment Parameters in Solid Tumor

Since the tissue thickness differed between the window chamber tumor model and the solid tumor model, the cycle number parameter for the 2 MHz HIFU transducer was adjusted to maintain the effectiveness of the anti-vascular effect induced by NB treatment in solid tumors (n = 12). A US image-guided HIFU treatment system integrating an 8 MHz imaging transducer with a 2 MHz HIFU transducer was used to record tumor perfusion images (Figure 1B). The flowchart of HIFU treatment and tumor perfusion tracing is shown in Figure 1C. Before treatment, mice were i.v. injected with 5 × 10^7^ NBs in 50 μL to acquire whole-tumor perfusion imaging under US contrast mode. Then, a 2 MHz HIFU transducer transmitted 1k, 2k, and 3k cycle US pulses at 2.6 MPa with 2 s intervals to scan half of the tumor for 10 min. The sonication time was defined as the moment when the intratumoral NB signals completely disappeared under HIFU scanning. This procedure was repeated once to complete the scanning of the entire tumor. The total treatment dose was 1 × 10^8^ NBs per mouse, with a total sonication time of 20 min. After treatment, mice were i.v. injected with 5 × 10^7^ NBs immediately and 1 h post-treatment to acquire whole-tumor perfusion imaging. The percentage of tumor perfusion was calculated by quantifying the intensity of NB signals within tumors using MATLAB software. The optimal parameters for 2 MHz HIFU in solid tumor treatment were determined through tumor perfusion tracing followed by subsequent histological assessments.

### 2.7. Histological Assessment of Tumor Necrosis and Vascular Destruction

The degree of the anti-vascular effect induced by NB and ONB treatment was compared through histological assessments (n = 20). The ONB treatment protocol followed the same procedure as the NB treatment. Mice were sacrificed 24 h after NB and ONB treatment. Tumors were excised and immersed in the optimal cutting temperature compound (Avantor, Radnor, PA, USA) overnight at −20 °C. The tumor was sectioned into 15 µm-thick frozen sections using a cryostat microtome (Cryotome E, Thermo Fisher Scientific, Sunnyvale, MA, USA). The hematoxylin and eosin (H&E) staining was used to define the necrosis area induced by the anti-vascular effect. Immunohistochemical staining for CD31 was used to evaluate vessel density. Tumor frozen sections were blocked for 40 min at room temperature. The sections were then incubated with a 1:100-diluted primary antibody (Purified rat anti-mouse CD31, BD Biosciences, Sparks, MD, USA) for 2 h, followed by incubation with a 1:100-diluted secondary antibody (Rabbit anti-rat IgG-FITC, Sigma-Aldrich, St. Louis, MO, USA) for 30 min. H&E and CD31-stained tumor section images were analyzed to quantify the necrotic area and vessel density under different cycle numbers of US pulses. The percentage of necrosis was calculated as the ratio of the necrotic area to the total tumor area. The percentage of vessel density was normalized to the mean vessel density of the control group. Intravital imaging, tumor perfusion imaging, and histological imaging were analyzed to determine the optimal US sonication parameters for ONB treatment-induced anti-vascular therapy.

### 2.8. Tumor Hypoxia Evaluation After ONB Treatment

To assess tumor reoxygenation after ONB treatment, mice (n = 12) were sacrificed 24 h after treatment and tumors were removed for enzyme-linked immunosorbent assay (ELISA). The tumors were cut into small pieces and placed in a single-cell tube (RWD Lifescience, Shenzhen, China), followed by the addition of 1 mL Dulbecco’s PBS (CORNING). The samples were homogenized using a single-cell suspension dissociator (DSC-400, RWD Lifescience, China) for 40 s. The suspension was collected after centrifugation at 5000× *g* for 5 min at 4 °C. The expression level of hypoxia inducible factor-1 alpha (HIF-1α) in the tumor suspensions was measured using a commercial ELISA kit (CUSABIO, Houston, TX, USA) and a plate reader system (SLXFA-SN, BioTek, Winooski, VT, USA), following the manufacturers’ protocols.

### 2.9. Detecting Immune-Related Cytokines in Tumors by ELISA

The feasibility of immune activation induced by ONB treatment was assessed on day 2 and day 4 after ONB treatment. Mice (n = 24) were sacrificed and tumors were removed to detect immune-related cytokines by ELISA. Tumors were homogenized following the above steps. Commercial ELISA kits (CUSABIO, Houston, TX, USA) were used to detect cytotoxic T lymphocyte-associated antigen-4 (CTLA-4), interferon gamma (IFN-γ), tumor necrosis factor-alpha (TNF-α), and interleukin-6 (IL-6). The expression of CTLA-4 represented immunosuppressive regulatory T cells, serving as an indicator of immune suppression. IFN-γ is produced by immune-enhancing T cells or natural killer cells. TNF-α and IL-6 are secreted by immune-activated cells, such as macrophages, T cells, and dendritic cells.

### 2.10. Detecting Immune Cells in Spleens by Flow Cytometry

The activation of systemic immunity induced by ONB treatment was evaluated by detecting immune cells in the spleens. On days 2 and 4 after ONB treatment, mice (n = 24) were sacrificed to collect spleens. The spleens were then cut into small pieces and gently pressed to isolate splenic immune cells for flow cytometry. The cell suspensions were centrifuged at 410× *g* for 5 min at 4 °C. The pellet was then mixed with 3 mL of RBC lysis buffer, and the reaction was stopped by adding 3 mL of Dulbecco’s modified Eagle’s medium. After centrifugation, the cells were resuspended in 1 mL of DPBS containing 2% fetal bovine serum. The samples were filtered through 70-µm cell strainers (BD Biosciences) to obtain single-cell suspensions.

To prevent non-specific binding, cells were blocked with azide-free Fc receptor blocking (Innovex Bioscience, Richmond, CA, USA) and stained with anti-CD45 (550994, BD Biosciences), anti-CD8 (553033, BD Biosciences), and anti-CD4 (110405, BioLegend, San Diego, CA, USA). The stained cells were analyzed using a BD Accuri C6 flow cytometer (Milpitas, CA, USA), and the data were processed with FlowJo software (version 1.0.23.1, BD Biosciences). CD45+CD4+ cells were identified as helper T cells, while CD45+CD8+ cells were identified as cytotoxic T cells.

### 2.11. Tumor Growth Tracing in Primary and Distant Tumors

Tumor growth in both primary and distant tumors was tracked to evaluate the effect of the systemic immune response induced by ONB treatment. A US imaging system was used to measure and calculate tumor volume. The primary tumors were treated with ONBs on days 0 and 4, and tumor size was monitored until day 14 (Figure 1D). On day 8 post-treatment, 1 × 10^6^ Panc02 cells in 100 μL were subcutaneously injected into the contralateral leg of the mice to establish distant tumors. These distant tumors were not treated and tumor size was monitored for 6 days. After completing the tumor growth tracking, mice were sacrificed and primary tumors were harvested for H&E staining to quantify the volume of necrosis.

### 2.12. Statistical Analysis

Both in vitro and in vivo data were obtained from at least three independent experiments, and the results are expressed as mean ± standard deviation. Statistical analyses were performed using SPSS software (version 24, International Business Machines Corporation, IBM, Armonk, NY, USA). The two-tailed, unpaired Student’s *t*-test was used for two-group comparisons. Multiple comparisons were made using one-way ANOVA with post hoc Bonferroni’s test. Statistically significant differences were considered for *p* < 0.05.

## 3. Results

### 3.1. Optimal Ratio of Gas Mixture for ONB Fabrication

**Figure 2:**
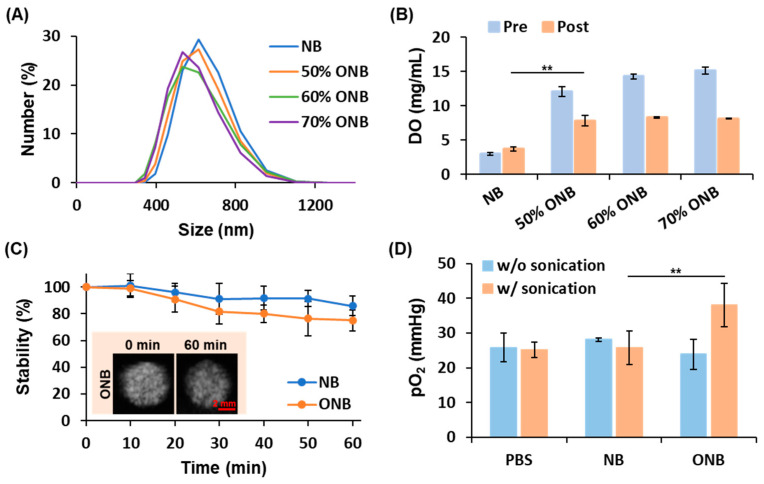
In vitro characteristics of ONBs: (**A**) size distribution; (**B**) pre- and post-washed DO levels; (**C**) the stability of ONBs tracing by US contrast imaging; (**D**) the pO_2_ levels of ONBs are increased to demonstrate the capability of oxygen release. (** *p* < 0.01).

The size distribution, concentration, and DO levels of ONBs were compared to those of NBs for determining the optimal oxygen ratios in the gas mixture for ONB fabrication. The size distribution curves show the population percentages at different size scales, demonstrating that the majority of ONBs fall within the size range of 400 to 1000 nm (Figure 2A). The size distributions of ONBs with different oxygen ratios show no significant difference from that of NBs. In the NB, 50% ONB, 60% ONB, and 70% ONB groups, the mean sizes were 639 ± 29, 630 ± 17, 597 ± 24, and 627 ± 65 nm, respectively, and the concentration of micro-sized bubbles were 5.8 ± 0.6, 4.1 ± 0.3, 3.5 ± 0.8, and 4.7 ± 1.4 × 10^10^ bubbles/mL, respectively. Compared to the NB, the fabrication of ONB by replacing 50–70% of octafluoropropane with oxygen had no effect on the size distribution, but slightly reduced the concentration, which remained acceptable for use.

The in vitro pre-washed DO levels were increased with the oxygen ratios in the gas mixture for ONB fabrication (Figure 2B). The post-washed DO levels of NBs, 50% ONBs, 60% ONBs, and 70% ONBs were 3.7 ± 0.3, 7.8 ± 0.8, 8.3 ± 0.1, and 8.1 ± 0.0 mg/mL, respectively. In the ONB groups, the post-washed DO levels were lower than the pre-washed DO levels, indicating that a portion of oxygen was dissolved in the solution. All ONB groups showed no significant difference in post-washed DO levels, suggesting the possibility of saturation in oxygen loading with ONBs. Therefore, 50% ONBs were chosen as ONBs for subsequent studies.

### 3.2. In Vitro Stability of ONBs

Ultrasound contrast images were used to evaluate the stability of NBs and ONBs. After 60 min, the stability was 85.8 ± 7.5% in the NB group and 75.1 ± 7.8% in the ONB group (Figure 2C). The results showed no significant differences between NBs and ONBs, demonstrating that carrying oxygen does not affect the stability of ONBs.

### 3.3. Oxygen Release from ONB Disruption

The capability of oxygen release from ONBs was estimated by detecting the in vitro pO_2_ levels. Before sonication, the pO_2_ levels showed no significant differences between the PBS, NB, and ONB groups (Figure 2D). The pO_2_ levels increased from 24 ± 4 to 38 ± 6 after sonication, which was observed only in the ONB group, demonstrating oxygen release from ONB disruption under sonication. 

### 3.4. In Vivo Hemorrhage Degree Induced by Anti-Vascular Effect

**Figure 3:**
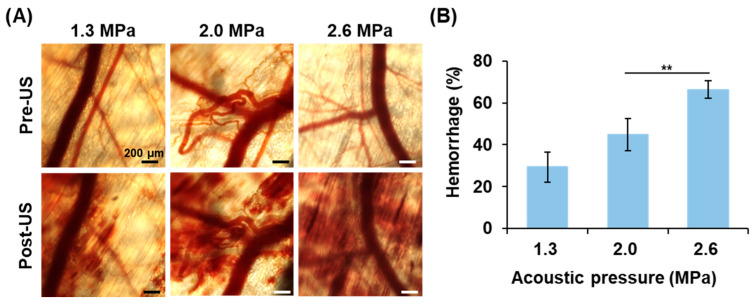
The degree of anti-vascular effect induced by NB treatment. (**A**) Intravital images show vascular disruption and hemorrhage after US stimulation. (**B**) The percentage of hemorrhage is proportional to the acoustic pressures. (each group n = 3; ** *p* < 0.01).

Intravital images were recorded to evaluate the anti-vascular effect induced by NB with US stimulation. In Figure 3A, the pre-US images show the intact vessel pattern, while post-US images reveal vessel disruption and hemorrhage. The areas of hemorrhage expanded with increasing acoustic pressures. The quantification of hemorrhage percentage was 29.4 ± 7.2, 44.8 ± 7.6, and 66.3 ± 4.2% under the 1.3, 2.0, and 2.6 MPa, respectively (Figure 3B). The highest acoustic pressure of 2.6 MPa demonstrated a significant enhancement in the anti-vascular effect, and this acoustic pressure will be used in subsequent solid tumor treatments.

### 3.5. Changes in Solid Tumor Microenvironment

**Figure 4:**
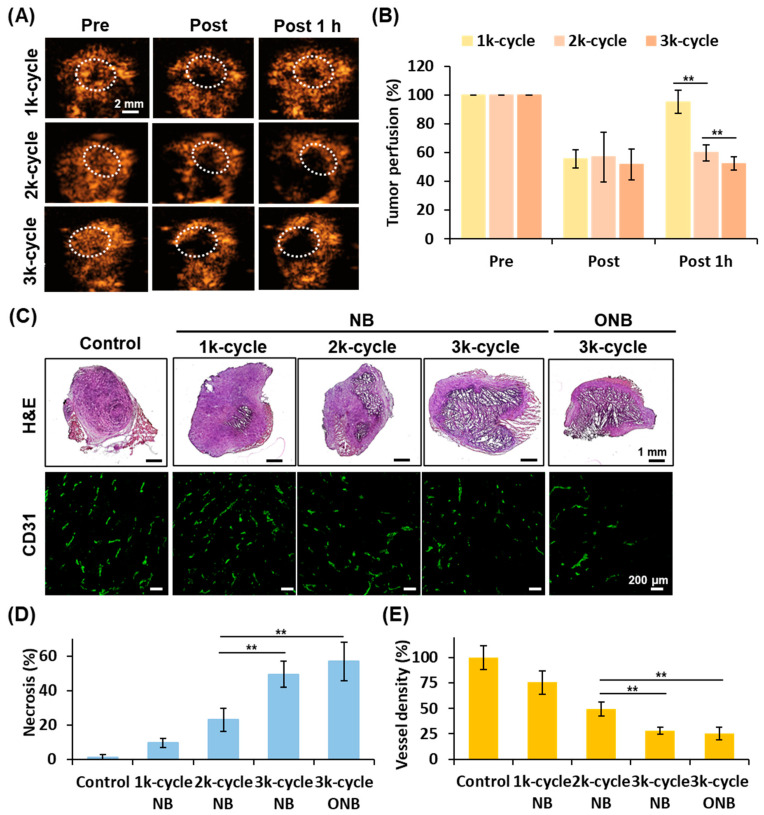
The evaluation of optimal US parameters for anti-vascular therapy. (**A**) Tumor perfusion tracing under the US contrast mode imaging. The dotted circle indicates tumor contour. (**B**) Quantification results of tumor perfusion tracing. (**C**) Histological images of H&E and CD31 staining. (**D**,**E**) Quantification data of tumor necrotic percentage and vessel density (each group n = 4; ** *p* < 0.01).

Considering that the thickness of solid tumors was greater than that of dorsal skinfold tumors, the cycle number of US parameters was adjusted to induce sufficient anti-vascular effect within solid tumors. US perfusion imaging showed high contrast enhancement in tumors before US stimulation, followed by reduced NB signals in post-imaging, indicating that the anti-vascular effect had occurred (Figure 4A). The post-1 h images revealed contrast recovery in the 1k cycle group due to vasoconstriction recovery, but no visible change in the 2k and 3k cycle groups. The quantified tumor perfusion results at post-1 h were 95 ± 8, 60 ± 6, and 52 ± 5% in the 1k, 2k, and 3k cycle groups, respectively (Figure 4B).

Histological assessments were conducted to evaluate the subsequent changes in the solid tumor microenvironment after the anti-vascular effect induced by NB and ONB treatment. In the H&E and CD31 staining, tumor necrosis areas expanded, and vessel density decreased with the increasing cycle number (Figure 4C). The percentage of necrosis in the control, 1k cycle NB, 2k cycle NB, 3k cycle NB, and 3k cycle ONB groups was 1.3 ± 1.5%, 9.6 ± 2.8%, 23.0 ± 6.8%, 49.5 ± 7.5%, and 57.0 ± 11.2%, respectively, while the corresponding vessel density was 100 ± 12%, 75 ± 11%, 49 ± 7%, 28 ± 3%, and 26 ± 3% (Figure 4D). These results demonstrated that the 3k cycle US parameters exhibited a relatively higher anti-vascular effect to induce necrotic areas within solid tumors. A comparison with NBs and ONBs under 3k cycle US stimulation showed no significant differences in tumor necrosis and vessel density.

### 3.6. Anti-Tumor Immune-Related Cytokine Expression

**Figure 5:**
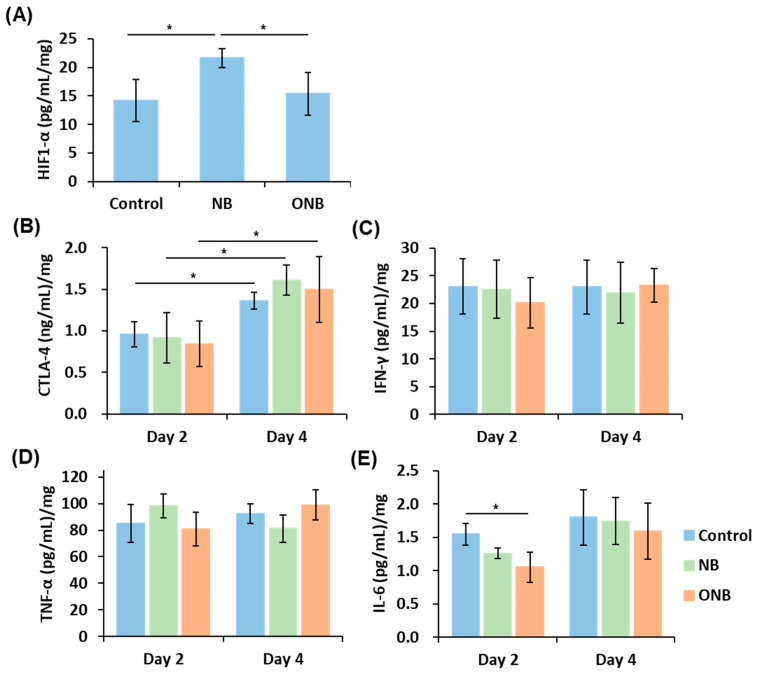
(**A**) Intratumoral protein expression of HIF-1α at 24 h after treatment. The expression levels of anti-tumor immune-related cytokines, including (**B**) CTLA-4, (**C**) IFN-γ, (**D**) TNF-α, and (**E**) IL-6. (each group, n = 4; * *p* < 0.05).

After 24 h treatment, tumor hypoxia was evaluated by ELISA since necrotic tumors were not suitable for hypoxia staining. The expression levels of HIF-1α were 14.2 ± 3.7, 21.6 ± 1.6, and 15.4 ± 3.7 pg/mL/mg in the control, NB, and ONB group, respectively (Figure 5A). The HIF-1α expression in the ONB group showed no significant difference from that in the control group (*p* = 0.888), demonstrating that ONB treatment compensated for oxygen supply to tumors during anti-vascular therapy.

In addition, the feasibility of activating anti-tumor immune responses was evaluated by analyzing the expression of immune-related cytokines in tumors. The expression levels of CTLA-4 significantly increased from day 2 to day 4 after treatment, but there were no significant differences between groups (Figure 5B). The expression levels of IFN-γ and TNF-α showed no significant changes in any group at day 2 and day 4 (Figure 5C,D). At day 2, the expression level of IL-6 in the ONB group was significantly lower than that in the control group, but no difference was observed at day 4 (Figure 5E).

### 3.7. Composition of Immune Cells in the Spleen

**Figure 6:**
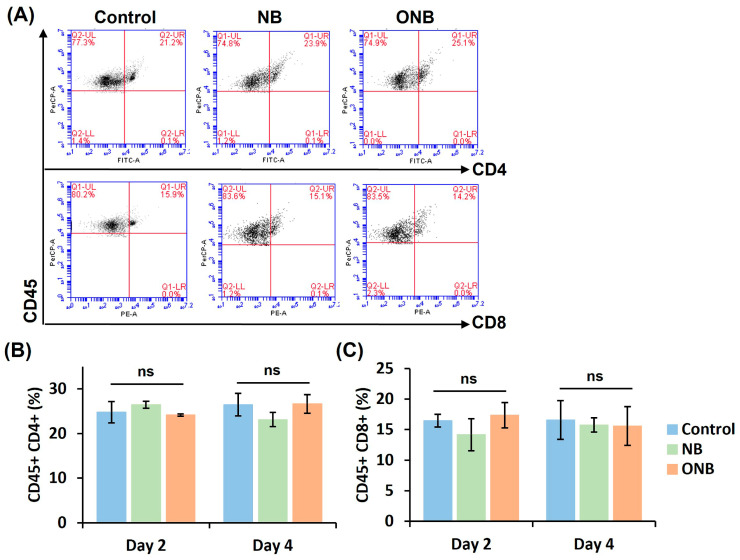
The composition of splenic immune cells detected by flow cytometry. (**A**) Representative flow cytometry plots showing the CD4+ and CD8+ T cells at day 4 after treatment. The percentage of (**B**) CD45 + CD4+ helper T cells and (**C**) CD45 + CD8+ cytotoxicity T cells in spleen at day 2 and day 4 after treatment. (in each group, n = 4; ns, non-significant difference *p* > 0.05).

Splenic immune cells were collected to quantify helper T cells and cytotoxic T cells, evaluating the feasibility of systemic anti-tumor immune activation. Figure 6A shows the flow cytometry results at day 4 after treatment. Immune cells expressing both CD45 and CD4 were defined as helper T cells, while those expressing both CD45 and CD8 were defined as cytotoxic T cells. There were no significant differences in immune cell composition between day 2 and day 4 across all groups. The percentage of CD45 + CD4+ T cells at day 4 was 26.5 ± 2.5, 23.2 ± 1.6, and 26.7 ± 2.1 in the control, NB, and ONB groups, respectively (Figure 6B), while the percentage of CD45 + CD8+ T cells was 16.6 ± 3.2, 15.8 ± 1.2, and 15.6 ± 3.2, respectively (Figure 6C). These quantification results revealed no significant differences in the detection of both helper T cells and cytotoxic T cells.

### 3.8. Primary and Distant Tumor Growth Tracing

**Figure 7:**
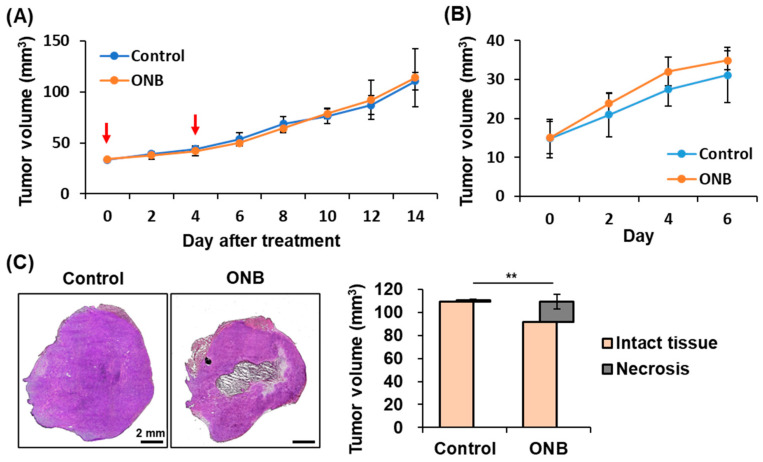
The growth curve of (**A**) primary tumors and (**B**) distant tumors. The red arrows indicate the treatment time. (**C**) H&E-stained images and quantification results of necrosis in primary tumors on day 14. (each group n = 5; ** *p* < 0.01).

Finally, tumor volumes were measured to evaluate the anti-tumor effect. The primary tumor growth curve illustrates the results of anti-vascular therapy induced by ONB treatment (Figure 7A). At day 14 after two-times treatment, tumor volume was 111 ± 9 and 114 ± 29 mm^3^ in the control and ONB groups, respectively. Meanwhile, the distant tumor growth curve was tracked to assess the activation of systemic immune responses (Figure 7B). Tumor growth tracing showed no significant reduction in either primary or distant tumors following ONB treatment. However, histological assessment revealed 17.3 ± 6.2 mm^3^ of necrosis within the tumors, which provide evidence for the anti-tumor effect induced by ONB treatment (Figure 7C).

## 4. Discussion

Anti-vascular therapy has been reported to activate anti-tumor immune responses by generating antigens from disrupted tumor vessels. However, the resulting tumor hypoxia due to insufficient vasculature might suppress immune cell function in immunotherapy [38]. Striking a balance between tumor vessel destruction and preservation is a crucial and complex issue in anti-vascular therapy-associated immunotherapy. Our study proposed that ONB treatment may locally compensate for oxygen in vessel-disrupted regions, preventing hypoxia-induced immunosuppression in combination tumor therapy. The optimal ONB formulation encapsulated 50% octafluoropropane and 50% oxygen, forming ONBs with a mean size of 630 ± 17 nm. In vitro pO_2_ levels increased by 14 ± 8 mmHg after oxygen release from ONBs, demonstrating their capability for oxygen delivery. For the in vivo studies, a tumor perfusion reduction threshold of 50%, along with a similar threshold for tumor necrosis, was used to determine the optimal US parameters for effective anti-vascular therapy. ONB treatment reduced tumor hypoxia by 29 ± 4% relative to the NB group, confirming its role in oxygen compensation. However, subsequent immune responses following ONB treatment showed no significant activation in cytokine expression or splenic immune cell composition. Although primary and distant tumor growth was not significantly inhibited in terms of volume, necrosis was observed in the center of the primary tumor to provide the efficacy of ONB treatment. After correcting tumor size based on the necrotic volume, the primary tumor volume following ONB treatment was reduced by 15.7 ± 5.0% compared to the control group.

The primary reasons of ineffective immune activation may include insufficient production of effective antigens, inadequate oxygen compensation, or unsuitable evaluation time points. Since the morphology of vessels differs between the tumor center and periphery, the uneven anti-vascular effect reduces the overall efficacy of tumor treatment [4,39,40]. In our previous study, HIFU parameters were adjusted to enhance the spatial uniformity of the anti-vascular effect and demonstrated that the degree of vascular disruption was primarily proportional to the acoustic pressures [20]. In the present study, we considered a 50% reduction in perfusion to be an appropriate degree of anti-vascular effect to activate the subsequent immune response. However, the therapeutic efficacy was not significant in terms of immune activation and distant tumor growth inhibition. These negative results highlighted a crucial issue regarding the complex correlations between vessel disruption, antigen production, oxygen delivery, hypoxia, and immunity when combining anti-vascular therapy with immunotherapy.

Our previous studies demonstrated that oxygen-loaded bubble treatment can significantly increase the in vivo pO_2_ levels in the murine solid tumor [28], murine hind–limb ischemia–reperfusion model [34], and murine ischemic stroke model [35]. However, the real-time changes in vivo pO_2_ level during ONB treatment were not detected due to the severe hemorrhage in solid tumors. Although the expression of HIF-1α in tumors was significantly reduced 6.22 ± 4.09 pg/mL/mg in the ONB group compared to the NB group 24 h after the first treatment, the destructive, hemorrhagic, and necrotic tumor microenvironment after anti-vascular effect inhibit the efficiency of ONB and oxygen delivery in the subsequent treatments. To address this issue, a priming reoxygenation prior to anti-vascular therapy could be a potential strategy to maintain the anti-tumor effect of vascular disruption and reduce the degree of tumor hypoxia. The first ONB treatment with low-intensity US could locally release oxygen without causing vessel damage, while the second ONB treatment with high-intensity US could disrupt tumor vessels to provide antigens for subsequent immune activation.

The enhancement of anti-tumor immune responses has been investigated at various time points following microbubble treatment. Although immune activation is typically a long-term effect, some studies have reported a significant increase in the population and infiltration of immune cells within a short-term observation period of 1 to 3 days post-treatment. Liu et al. used a murine colon tumor model to investigate the antitumor immune response induced by increased permeability of the tumor microvasculature under microbubble cavitation [41]. The population of cytotoxic T cells significantly and progressively increased over time on days 1, 3, and 18 in the treated group compared to the control. However, no significant differences were observed in the population of helper T cells. Tang et al. used an invasive US needle to induce anti-tumor immune responses in a murine colorectal tumor model through mechanical destruction [42]. Significant increases in the infiltration of dendritic cells and helper T cells within tumors were observed on day 3 after treatment. On the other hand, some studies employed long-term tracing to evaluate the activation of immune responses, but the results showed inconsistent outcomes. Wu et al. used low-intensity-focused US with microbubbles to enhance immune activation in murine 4T1 tumor model [43]. After 5 consecutive days of treatment, tumor growth was significantly inhibited and activated immune responses on day 7. The proportion of mature dendritic cells and cytotoxicity T cells in tumor-draining lymph nodes were significantly increased. Abe et al. reported that the mechanical effect of HIFU significantly enhanced the infiltration of helper T cells and cytotoxic T cells into tumors on day 13 after treatment [30]. Bulner et al. evaluated immune activation in murine colon tumors following microbubble cavitation [25]. Tumor perfusion was reduced by 88 ± 3.6% after microbubble cavitation to demonstrate an anti-vascular effect. However, immune cell populations in the tumors and tumor-draining lymph nodes showed no significant increase on days 3 and 7 after treatment. Based on the above results, US-stimulated microbubble cavitation may not be sufficient to cause a significant enhancement in anti-tumor immune activation. The additional administration of immune checkpoint inhibitors, such as anti-PD-L1, resulted in more improvements in immune responses compared to microbubble cavitation alone [25,30,42,44].

In immunotherapy, tumor immunosuppressive microenvironments inhibit the efficacy of anti-tumor immune cells [3,45]. Pancreatic ductal adenocarcinoma is classified as an immunologically “cold” phenotype, characterized by limited immune cell infiltration, an immune-suppressive tumor microenvironment, and low immunogenicity [46]. Although sufficient antigens are generated from the disrupted tumor vasculature, the limited and poorly recognized antigens on tumor cells may hinder the effectiveness of immune activation [47]. On the other hand, although the anti-vascular effect could provide effective antigens to activate immune cells, the infiltration and function of activated immune cells within tumors may still be limited due to abnormal vasculature, poor blood perfusion, high solid stress, and hypoxic conditions [48]. Thus, reversing the tumor microenvironment from immunosuppression to immunoactivation is a key strategy to activate anti-tumor immune cells for tumor elimination. US-stimulated bubble cavitation not only disrupts vessels, but also increases vascular permeability, induces vasodilation, and enhances blood perfusion to remodel the tumor microenvironment and improve the efficacy of immunotherapy [49]. Li et al. proposed that enhancing tumor perfusion via microbubble cavitation could increase the infiltration of helper T cells in tumors [50]. Acoustic pressures of 0.8 MPa and 2.4 MPa were applied to induce vessel dilation and disruption, respectively. Tumor perfusion was significantly increased in the 0.8 MPa group, while intratumoral vessel damage and hemorrhage were observed in the 2.4 MPa group. At 24 h after treatment, the percentages of infiltrating helper T cells in tumors were 32.35%, 19.43%, and 26.03% in the 0.8 MPa, 2.4 MPa, and control groups, respectively. These results indicated that enhancing tumor perfusion without vascular disruption may be a promising strategy to promote anti-tumor immune responses.

## 5. Conclusions

Our study evaluated the potential of ONB treatment in combining local oxygen release and anti-vascular therapy to activate anti-tumor immunity. However, immune activation following ONB treatment was not significantly observed in the spleens and tumors, possibly due to the imbalance between vessel destruction and preservation. These findings highlight the need for further investigation into the complex interactions between antigen production, oxygen delivery, tumor hypoxia, and immune function in anti-vascular therapy-associated immunotherapy.

## Data Availability

The original contributions presented in this study are included in the article. Further inquiries can be directed to the corresponding author.

## Abbreviations

US	Ultrasound
NB	Nanobubble
HIFU	High-intensity focused ultrasound
ONB	Oxygen-loaded nanobubble
DO	Dissolved oxygen
PBS	Phosphate-buffered saline
pO_2_	Partial pressure of oxygen
Panc02	Murine pancreatic ductal adenocarcinoma
H&E	Hematoxylin and eosin
ELISA	Enzyme-linked immunosorbent assay
HIF-1α	Hypoxia inducible factor-1 alpha
CTLA-4	Cytotoxic T lymphocyte-associated antigen-4
IFN-γ	Interferon-gamma
TNF-α	Tumor necrosis factor-alpha
IL-6	Interleukin-6
